# *Spiked Helmet Sign* : Um Caso Atípico de Supradesnivelamento Transitório do Segmento ST no ECG

**DOI:** 10.36660/abc.20201017

**Published:** 2021-06-08

**Authors:** Acácio F. Cardoso, Marco Alexander V. Akamine, Rafael M. Pessoa, Elizabeth T. Takitani, José V. Kairiyama, Manfredo K. Naritoni

**Affiliations:** 1 Serviço de Cardiologia Hospital Nipo-Brasileiro São PauloSP Brasil Serviço de Cardiologia do Hospital Nipo-Brasileiro , São Paulo , SP - Brasil

**Keywords:** Supradesnivelamento-ST, Eletrocardiografia/métodos, Ecocardiografia/métodos, Morte Súbita Cardíaca, Doença Grave

## Apresentação do caso

**Figura 1:**
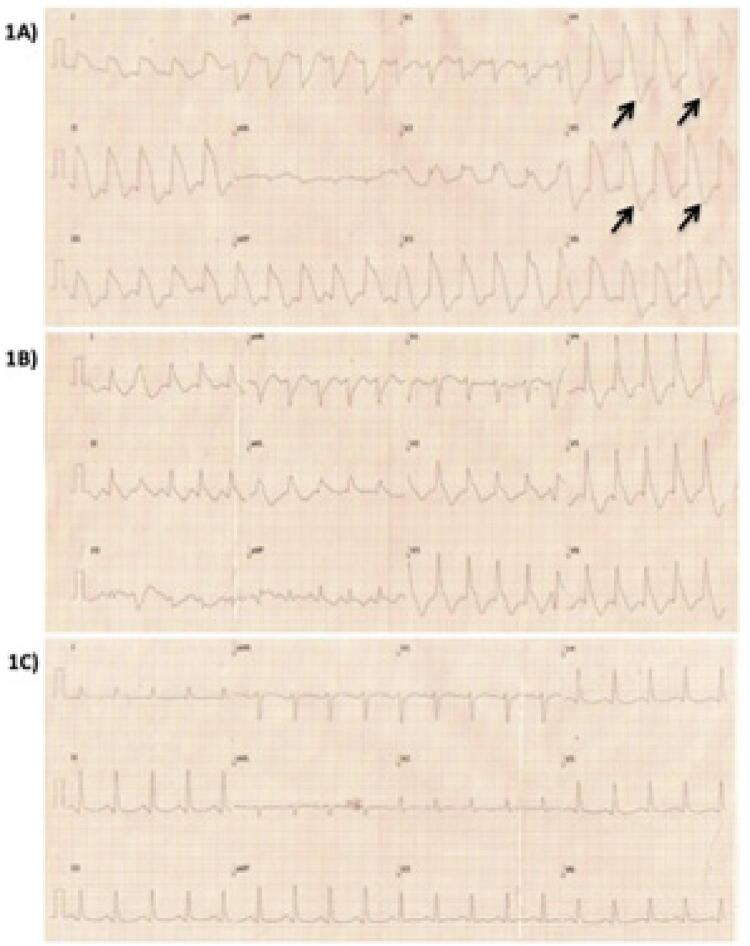
– Sequência de ECGs da admissão do paciente. 1A) ECG inicial com supradesnivelamento atípico de ST em múltiplas derivações seguidas de macroalternância das ondas T facilmente observadas em V4 e V5 (setas). 1B) Uma hora após o ECG inicial, um novo registro demonstrou uma redução de aproximadamente 50% do supradesnivelamento de ST. Os achados característicos do SHS tornam-se mais evidentes nas derivações V2 e V3. 1C) Seis horas após o ECG inicial, nota-se a resolução completa do supradesnivelamento de ST com a manutenção apenas de alterações discretas da repolarização ventricular.

Homem, 35 anos de idade, tabagista e usuário de drogas ilícitas (maconha, cocaína e solventes inalantes), em tratamento com antipsicótico e antidepressivo (haloperidol e escitalopram), há dois dias com vários episódios de vômitos e diarreia, evoluiu com confusão mental no domicílio e foi admitido na sala de emergência com rebaixamento do nível de consciência e respiração irregular. Logo em seguida, apresentou quadro de parada cardiorrespiratória (PCR) com registro de fibrilação ventricular (FV) no monitor cardíaco. Após seis minutos de manobras de ressuscitação cardiopulmonar, infusão de adrenalina e duas desfibrilações cardíacas, o pulso e o ritmo cardíaco foram recuperados. Realizou-se eletrocardiograma (ECG), que demonstrou elevação atípica e difusa do segmento ST ( Figura 1 A). Os exames laboratoriais iniciais demonstraram acidose metabólica grave com nível sérico elevado de lactato (lactato sérico = 26 mg/dl), além de hipernatremia (sódio sérico = 153 mg/dl), hipocalemia (potássio sérico = 3,2 mg/dl), leucocitose importante e discreto aumento da troponina sérica. O paciente foi entubado e colocado em ventilação mecânica. Realizou-se ressuscitação volêmica, além de correção da acidose metabólica, e antibióticos de largo espectro foram iniciados. Após uma hora do atendimento inicial, realizou-se novo ECG, que demonstrou uma redução de aproximadamente 50% no supradesnivelamento de ST ( Figura 1B ). Dado o caráter atípico das alterações do segmento ST no ECG, optou-se por não realizar coronariografia de urgência. Seis horas após o evento, as alterações iniciais do ECG regrediram completamente ( Figura 1C ). Realizou-se ecocardiograma, que demonstrou disfunção importante do ventrículo esquerdo às custas de hipocinesia difusa (fração de ejeção = 0,36). Fez-se novo ecocardiograma dois dias após, que revelou completa recuperação da função ventricular (fração de ejeção = 0,69). Realizou-se angiotomografia de coronárias durante a internação, que demonstrou ausência de lesões obstrutivas e descartou anomalias coronarianas. O paciente evoluiu bem e recebeu alta após oito dias de internação.

## Discussão

Neste intrigante relato de caso, um paciente jovem, usuário de drogas ilícitas, em uso regular de haloperidol e escitalopram, deu entrada no setor de emergência em uma condição clínica grave, em iminência de PCR. Após episódio de FV prontamente revertida, o ECG inicial demonstrou ritmo taquicárdico, com complexos QRS alargados, aparentemente precedidos de ondas P de baixa amplitude. A ativação inicial do QRS era rápida e acompanhada de supradesnivelamento de ST com morfologia convexa em múltiplas derivações eletrocardiográficas, seguidas da inversão e alternância da amplitude das ondas T, fenômeno conhecido por macroalternância das ondas T ( Figura 1A ).

O sinal do capacete pontiagudo, tradução para *spiked helmet sign* (SHS), foi descrito por Littmann et al. ^1^ em 2011 como uma elevação transitória do segmento ST em condições clínicas graves de origem não cardíaca, associada a níveis séricos normais ou pouco aumentados de troponina, além de evolução clínica desfavorável com alta taxa de mortalidade. ^1^ Na sua série, 6 de 8 pacientes morreram após o registro inicial do ECG dentro de 1 a 10 dias. ^1^ Inicialmente descrito como uma elevação do segmento ST restrita às derivações inferiores, novos casos têm sido relatados com envolvimento de múltiplas derivações eletrocardiográficas. ^2^ A morfologia no ECG assemelha-se ao *pickelhaube* , um capacete cravejado por uma haste pontiaguda, utilizado por militares do exército da Prússia e Alemanha durante os séculos XIX e XX.

**Figura 2:**
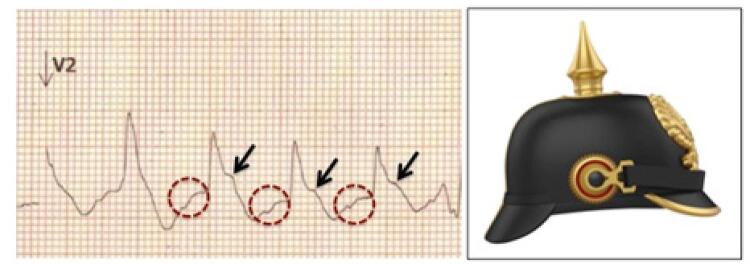
– Principais achados do SHS no ECG. Uma linha isoelétrica ascendente (círculos vermelhos) é seguida por supradesnivelamento convexo de ST (setas). As alterações se assemelham ao capacete pontiagudo utilizado pelos exércitos da Prússia e Alemanha nos séculos XIX e XX. Legenda: SHS – Spiked helmet sign.

As principais características do SHS no ECG são uma elevação ascendente da linha isoelétrica que precede o QRS, seguida por uma onda R estreita e uma elevação convexa do segmento ST ^2^ ( Figura 2 ). Os mecanismos fisiopatológicos relacionados a esse padrão morfológico no ECG ainda não estão totalmente esclarecidos. Uma onda T-U gigante prévia que avança sobre o próximo QRS e/ou o prolongamento da repolarização ventricular sobreposto a frequências cardíacas elevadas são possíveis causas atribuídas por alguns autores. ^3^ Os casos iniciais foram identificados em patologias torácicas e abdominais e associados a artefatos musculares e aumento agudo da pressão nessas cavidades. Posteriormente, outros relatos envolvendo hemorragia intracerebral, alterações metabólicas graves e choque séptico apontaram para uma intensa descarga adrenérgica como uma via final comum para o desencadeamento dessas alterações no ECG. Manifestações clínicas associadas a estados hiperadrenérgicos como após a ablação do gânglio estrelado ^4^ e na cardiomiopatia de Taktsubo ^5^ reforçam essa hipótese.

A macroalternância da onda T é uma manifestação rara no ECG, reflete uma importante dispersão da repolarização ventricular e geralmente precede o desencadeamento da FV. ^6^ Mais comumente, esse padrão morfológico é visto em pacientes com síndrome do QT longo congênito ou adquirido de alto risco e anuncia o início de uma *Torsade de Pointes* . No nosso caso, a macroalternância da onda T foi observada após uma FV abortada e indica que em manifestações extremas do *SHS* a repolarização ventricular pode se apresentar de forma bastante prolongada e propiciar o desencadeamento de arritmias ventriculares potencialmente fatais. Particularmente em pacientes psiquiátricos, essas alterações podem ser exacerbadas pelo uso de antipsicóticos e antidepressivos, drogas que sabidamente podem prolongar o potencial de ação da célula cardíaca ao promover o bloqueio das correntes iônicas de potássio. ^7^ Publicação prévia do SHS envolvendo o prolongamento do QT, alternância da onda T e *Torsade de Pointes* indicam que o SHS pode ser um possível mecanismo de morte súbita nesses pacientes. ^8^ Dada a raridade do fenômeno, a relação entre o SHS e o risco de morte súbita ainda precisa ser estabelecida em futuras publicações.

Outras situações clínicas associadas à elevação do segmento ST como bloqueios de ramo, pericardites, embolia pulmonar maciça e principalmente as síndromes coronarianas agudas devem ser consideradas como os principais diagnósticos diferenciais do SHS. ^9^ No nosso caso, o vasoespasmo coronariano associado ao abuso de cocaína e a desfibrilação cardíaca são outras duas situações que envolvem anormalidades da repolarização ventricular e devem ser consideradas nessa análise. No primeiro caso, a cocaína, além de precipitar episódios de vasoespasmo coronariano, pode atuar como potente inibidor das correntes dos canais iônicos responsáveis pelo potencial de ação da célula cardíaca. Ambas as condições podem favorecer o prolongamento da repolarização ventricular e o desencadeamento de arritmias ventriculares graves. ^10^ Entretanto, o vasoespasmo coronariano geralmente é precedido de dor torácica, as alterações do segmento ST costumam ser restritas a algumas derivações, dura apenas poucos minutos e são seguidas por ondas T simétricas e amplas no ECG. ^11^ Já a elevação do segmento ST associada a desfibrilação elétrica é um fenômeno de curta duração, atinge a sua máxima amplitude logo após o choque e tem média de duração de aproximadamente 60 segundos, retornando ao padrão normal em torno de 5 minutos após o choque. ^12^ Embora não seja possível excluir totalmente a participação dessas duas condições nas alterações evidenciadas no ECG, essas observações tornam essas hipóteses menos prováveis.

Alterações metabólicas e eletrolíticas são comuns em pacientes graves e podem apresentar manifestações eletrocardiográficas semelhantes às observadas no SHS. A acidose metabólica associada à hipercalemia grave frequentemente aumenta a duração do QRS e provoca supradesnivelamentos do segmento ST principalmente em precordiais direitas, sendo facilmente confundidos com infarto agudo do miocárdio de parede anterior. ^13^ A hipocalcemia acentuada é outra condição metabólica que pode provocar supradesnivelamento de ST e, juntamente com a hipocalemia, pode prolongar o intervalo QT de forma significativa. ^14^ Já mudanças no ECG relacionadas aos níveis séricos de sódio são mais raras. Infradesnivelamento de ST e encurtamento do intervalo PR já foram descritos em casos extremos de hipernatremia. ^15^ No nosso caso, acidose metabólica grave, hipernatremia e hipocalemia leve foram as únicas anormalidades metabólicas identificadas nos exames laboratoriais.

Esses achados não são suficientes para explicar todas as alterações observadas na sequência dos ECGs, o que torna o SHS uma manifestação eletrocardiográfica única com prognóstico, na maioria das vezes, bastante adverso.

## Conclusões

O SHS é uma rara manifestação no ECG de pacientes graves com patologias não cardíacas. O prolongamento da repolarização ventricular associada ao aparecimento de macroalternância das ondas T parece ser um mecanismo plausível de arritmias ventriculares malignas neste cenário e requer pronto reconhecimento e intervenção.
