# Supplementary material for: Spiked Helmet Sign : Um Caso Atípico de Supradesnivelamento Transitório do Segmento ST no ECG
Source: Arq Bras Cardiol. 2021 Jun 8;116(6):1165–8. [Article in Portuguese] doi: 10.36660/abc.20201017 (PMC8288532; doi:10.36660/abc.20201017)
Supplement: Translation

# Translation: Spiked Helmet Sign: An Atypical Case of Transient ST-Segment Elevation on ECG

**Authors:** Acácio F. Cardoso, Marco Alexander V. Akamine, Rafael M. Pessoa, Elizabeth T. Takitani, José V. Kairiyama, Manfredo K. Naritoni

**Affiliations:** 1 Brazil Serviço de Cardiologia do Hospital Nipo-Brasileiro, São Paulo, SP - Brazil

**Keywords:** ST-Elevation, Electrocardiography/methods, Echocardiography/methods, Death, Sudden Cardiac, Critical Illness

## Case report

Male, 35 years old, smoker, user of illicit drugs (marijuana, cocaine and inhalant solvents), on antipsychotics and antidepressants (haloperidol and escitalopram), several episodes of vomiting and diarrhea for the past two days, mental confusion at home, and admitted to the emergency room with reduced level of consciousness and irregular breathing. Soon afterwards, he presented cardiopulmonary arrest (CRP) with ventricular fibrillation (VF) on the cardiac monitor. After six minutes of cardiopulmonary resuscitation maneuvers, adrenaline infusion and two cardiac defibrillations, pulse and heart rate were recovered. An electrocardiogram (ECG) was performed, which demonstrated atypical and diffuse ST-segment elevation ( Figure 1 A ). Initial laboratory tests showed severe metabolic acidosis with high serum lactate (serum lactate = 26 mg/dl), in addition to hypernatremia (serum sodium = 153 mg/dl), hypokalemia (serum potassium = 3.2 mg/dl), severe leukocytosis and a slight increase in serum troponin. The patient was intubated and placed on mechanical ventilation. Volume resuscitation was performed, metabolic acidosis was corrected, and broad-spectrum antibiotics were initiated. One hour after initial consultation, a new ECG was performed, which demonstrated a reduction of approximately 50% in ST elevation ( Figure 1B ). Given the atypical character of the ST segment abnormalities on ECG, we chose not to perform an emergency coronary angiography. Six hours after the event, the initial ECG abnormalities receded completely ( Figure 1C ). An echocardiogram was performed, which showed severe left ventricular dysfunction at the expense of diffuse hypokinesia (ejection fraction = 0.36). A new echocardiogram was performed two days later, which revealed complete recovery of ventricular function (ejection fraction = 0.69). Coronary angiotomography was performed during hospitalization, which demonstrated the absence of obstructive lesions and ruled out coronary anomalies. The patient progressed well and was discharged after eight days of hospitalization.

**Figure 1:** – Sequence of ECG on patient admission. 1A) Initial ECG with atypical ST-segment elevation in multiple leads followed by T-wave macroalternation easily observed in V4 and V5 (arrows). 1B) One hour after the initial ECG, a new scan showed a reduction of approximately 50% in ST-segment elevation. The characteristic findings of SHS are more evident in leads V2 and V3. 1C) Six hours after the initial ECG, complete resolution of ST-segment elevation is seen with only mild ventricular repolarization abnormalities.

## Discussion

In this intriguing case report, a young patient, an user of illicit drugs, on regular use of haloperidol and escitalopram, was admitted to the emergency department in a serious clinical condition, on the verge of a cardiac arrest. After a VF episode was promptly reversed, the initial ECG demonstrated tachycardic rhythm, with enlarged QRS complexes, apparently preceded by low amplitude P waves. Initial activation of QRS was rapid and accompanied by ST elevation with convex morphology in multiple electrocardiographic leads, followed by inversion and alternation of the amplitude of T waves, a phenomenon known as macroalternation of T waves (Figure 1A).

Spiked helmet sign (SHS) was described by Littmann et al.^1^ in 2011 as a transient ST-segment elevation in severe clinical conditions of non-cardiac origin, associated with normal or slightly increased serum levels of troponin, in addition to an unfavorable clinical outcome with a high mortality rate.^1^ In their series, 6 of 8 patients died after an initial ECG within 1 to 10 days.^1^ Initially described as ST segment elevation restricted to lower leads, new cases have been reported involving multiple electrocardiographic leads.^2^ Morphology on ECG is similar to the pickelhaube, a helmet studded with a pointed rod, worn by military Prussian and German army personnel in the 19^th^and 20^th^centuries.

The main SHS characteristics on the ECG are an upward elevation of the isoelectric line that precedes the QRS, followed by a narrow R wave and a convex ST-segment elevation^2^ ( figure 2 ). The pathophysiological mechanisms related to this ECG morphological pattern are not yet fully understood. A previous giant T-U wave that advances over the next QRS and/or prolongation of ventricular repolarization superimposed at high heart rates are potential causes attributed by some authors.^3^ Initial cases were identified in thoracic and abdominal pathologies and are associated with muscle artifacts and acute pressure increase in these cavities. Subsequently, other reports involving intracerebral hemorrhage, severe metabolic abnormalities and septic shock pointed to an intense adrenergic discharge as a common final route for triggering these ECG abnormalities. Clinical manifestations associated with hyperadrenergic states such as after stellate ganglion^4^ ablation and Taktsubo^5^ cardiomyopathy reinforce this hypothesis.

**Figure 2:** – Main SHS findings on ECG. An upward isoelectric line (red circles) is followed by convex ST-segment elevation (arrows). The abnormal findings are similar to the spiked helmet worn by the armies of Prussia and Germany in the 19th and 20th centuries. Key: SHS – Spiked helmet sign.

T-wave macroalternation is a rare ECG manifestation; it reflects severe dispersion of ventricular repolarization and generally precedes the onset of VF.^6^ This morphological pattern is more commonly seen in patients with congenital or acquired high-risk QT syndrome and announces the beginning of Torsade de Pointes. In our case, the T-wave macroalternation was observed after an aborted VF and indicates that in extreme manifestations of SHS, ventricular repolarization may last for a very prolonged time and lead to the onset of potentially fatal ventricular arrhythmias. Particularly in psychiatric patients, these abnormalities may be exacerbated by antipsychotics and antidepressants, which are known to prolong the cardiac cell action potential by blocking potassium ion currents.^7^ Previous publication of SHS involving QT prolongation, T-wave alternation and Torsade de Pointes has found that SHS may be a possible mechanism of sudden death in these patients.^8^ Given the rarity of the phenomenon, the relationship between SHS and the risk of sudden death is yet to be established in future publications.

Other clinical situations associated with ST-segment elevation, such as bundle branch block, pericarditis, massive pulmonary embolism, and especially acute coronary syndromes, should be considered as the main differential diagnoses of SHS.^9^ In our case, coronary vasospasm associated with cocaine abuse and cardiac defibrillation are two other situations that involve abnormalities in ventricular repolarization and should be considered in this analysis. In the first case, cocaine precipitates episodes of coronary vasospasm and can act as a potent inhibitor of ion channel currents responsible for the cardiac cell action potential. Both conditions may promote the prolongation of ventricular repolarization and the onset of severe ventricular arrhythmias.^10^ However, coronary vasospasm is usually preceded by chest pain, ST-segment abnormalities are usually restricted to some leads, last only a few minutes, and are followed by symmetrical and wide T-waves on ECG.^11^ ST-segment elevation associated with electrical defibrillation is a short-term phenomenon, it reaches its maximum amplitude right after the shock and has an average duration of approximately 60 seconds, returning to the normal pattern around 5 minutes after the shock. ^12^ Although it is not possible to totally rule out the participation of these two conditions in the abnormalities evidenced in the ECG, these findings make these hypotheses less likely.

Metabolic and electrolytic abnormalities are common in critically ill patients and may have electrocardiographic manifestations similar to those observed in SHS. Metabolic acidosis associated with severe hyperkalaemia often increases QRS duration and causes ST-segment elevation mainly in the right precordial leads, and is easily confused with acute anterior wall myocardial infarction.^13^ Marked hypocalcemia is another metabolic condition that may cause ST-segment elevation and, with hypokalemia, may significantly prolong the QT interval.^14^ ECG abnormalities related to serum sodium levels are rarer. ST-segment depression and shortening of the PR interval have been described in extreme cases of hypernatremia.^15^ In our case, severe metabolic acidosis, hypernatremia and mild hypokalemia were the only metabolic abnormalities identified in laboratory tests.

These findings are not sufficient to explain all the abnormalities observed in the sequence of ECG, which makes SHS a unique electrocardiographic manifestation with quite adverse prognosis in most cases.

## Conclusions

SHS is a rare manifestation on the ECG of critically ill patients with non-cardiac pathologies. Prolongation of ventricular repolarization associated with T-wave macroalternation seems to be a plausible mechanism of malignant ventricular arrhythmias in this scenario and requires prompt recognition and intervention.
